# Symptoms of Common Mental Disorders and Adverse Health Behaviours in Male Professional Soccer Players

**DOI:** 10.1515/hukin-2015-0130

**Published:** 2015-12-30

**Authors:** Vincent Gouttebarge, Haruhito Aoki, Gino Kerkhoffs

**Affiliations:** 1 World Players’ Union (FIFPro), Hoofddorp, The Netherlands; 2 Department of Orthopaedic Surgery, Academic Medical Center, Amsterdam, The Netherlands; 3 Academic Center for Evidence based Sports medicine (ACES), Academic Medical Center, Amsterdam, The Netherlands; 4 Amsterdam Collaboration for Health & Safety in Sports (ACHSS), Academic Medical Center / VU University Medical Center, Amsterdam, The Netherlands; 5 St. Marianna University School of Medicine, Kawasaki, Japan

**Keywords:** soccer, distress, anxiety, depression, sleeping disturbance, adverse health behaviours

## Abstract

To present time, scientific knowledge about symptoms of common mental disorders and adverse health behaviours among professional soccer players is lacking. Consequently, the aim of the study was to determine the prevalence of symptoms of common mental disorders (distress, anxiety/depression, sleep disturbance) and adverse health behaviours (adverse alcohol behaviour, smoking, adverse nutrition behaviour) among professional soccer players, and to explore their associations with potential stressors (severe injury, surgery, life events and career dissatisfaction). Cross-sectional analyses were conducted on baseline questionnaires from an ongoing prospective cohort study among male professional players. Using validated questionnaires to assess symptoms of common mental disorders and adverse health behaviours as well as stressors, an electronic questionnaire was set up and distributed by players’ unions in 11 countries from three continents. Prevalence of symptoms of common mental disorders and adverse health behaviours among professional soccer players ranged from 4% for smoking and 9% for adverse alcohol behaviour to 38% for anxiety/depression and 58% for adverse nutrition behaviour. Significant associations were found for a higher number of severe injuries with distress, anxiety/depression, sleeping disturbance and adverse alcohol behaviour, an increased number of life events with distress, sleeping disturbance, adverse alcohol behaviour and smoking, as well as an elevated level of career dissatisfaction with distress, anxiety/depression and adverse nutrition behaviour. Statistically significant correlations (p<0.01) were found for severe injuries and career dissatisfaction with most symptoms of common mental disorders. High prevalence of symptoms of common mental disorders and adverse health behaviours was found among professional players, confirming a previous pilot-study in a similar study population.

## Introduction

In the particular occupational category that is professional soccer, the overall level of injury in players has been shown to be around 1000 times higher than for other industrial occupations generally regarded as high-risk (Hawkins and Fuller, 1999). Professional soccer players are highly at risk for acute, recurrent and severe injuries during their career, severe injuries leading to both surgeries and long periods without training or competition (Aoki et al., 2012; Chen et al., 2005; Ekstrand at al., 2011; Gouttebarge et al., 2015b). In contrast to the extensive amount of information related to the physical health of players, scientific information about the occurrence of symptoms of common mental disorders (CMD) and adverse health behaviours is scarce in professional soccer (Krueger et al., 1998; Gouttebarge and Aoki, 2014). Symptoms of CMD can be classified in 20 different domains, which include symptoms related to depression, anxiety, sleep disorder, neurocognition and substance abuse/addiction (American Psychiatric Association, 2000). To present time, only one study has reported that symptoms of CMD and adverse health behaviours were highly prevalent among male professional soccer players (Gouttebarge et al., 2015a). Such limited evidence is peculiar as it has been shown among elite athletes from other sport disciplines that severe injuries and surgeries that occur during a sport career can be considered as major stressors that may induce symptoms of CMD (Shuer and Dietricht, 1997; Walker et al., 2007). Consequently, empirical studies focussing on the occurrence of symptoms of CMD and adverse health behaviours among professional players seem particularly needed, while one might assume that these outcomes might be the consequence of the occurrence of severe injuries and surgeries during a soccer career (Shuer and Dietricht, 1997; Walker et al., 2007). Even more, next to these sports-related stressors, professional players are just as likely as anyone to develop symptoms of CMD and adverse health behaviours as a consequence of more conventional stressors: theoretical models have emphasised that life dissatisfaction as well as major life events might lead to the occurrence of symptoms of CMD (Karasek and Theorell, 1990).

With regard to the aforementioned arguments, the aim of the present study was twofold: to determine the prevalence of symptoms of CMD (distress, anxiety/depression, sleep disturbance) and adverse health behaviours (adverse alcohol behaviour, smoking, adverse nutrition behaviour) among male professional soccer players, and to explore their associations with severe injury, surgery, life events and career dissatisfaction. Next to the hypothesis that symptoms of CMD and adverse health behaviours were substantially prevalent among players, it was assumed that a higher number of severe injuries, surgeries, life events or a higher level of career dissatisfaction were associated with the occurrence of symptoms of CMD and adverse health behaviours among male professional soccer players.

## Material and Methods

### Design, setting and participants

Reported in compliance with the STROBE statement, the present study is a cross-sectional analysis of the baseline questionnaires from an ongoing prospective cohort study (Vandenbroucke et al., 2007). Official approval of our study was issued by the board of the St. Marianna University School of Medicine (2014/04/16; Kawasaki, Japan). The present research was conducted in accordance with the Declaration of Helsinki (2013).

Participants were active professional soccer players. Inclusion criteria were as follows: (i) being a member of a national players’ union, which means committing significant time to soccer training and competing at a professional level; (ii) being aged 18 years old or older; (iii) being male; and (iv) being reading-comprehension fluent either in English, French, Japanese or Spanish. Sample size calculation indicated that at least 138 participants were needed (power of 80%; confidence interval of 95%; precision of 5%) under the assumption that one out of 10 players might suffer from a mental health condition (Woodward, 2013). Expecting a response rate of at least 25%, we intended to contact at least 560 players.

### Symptoms of common mental disorders and adverse health behaviours

#### Distress

Distress in the preceding four weeks was measured using the Distress Screener (3 items scored on a 3-point scale) which is based on the four-dimensional symptom questionnaire (4DSQ) (e.g., ‘Have you recently suffered from worry?’) (Braam et al., 2009). The 4DSQ *i.e.* Distress Screener had been validated in several languages including English, French and Spanish (internal consistency: 0.6 – 0.7; test-retest coefficients ≥ 0.89; criterion-related validity: Area Under ROC Curve ≥ 0.79) (Braam et al., 2009; Terluin et al., 2006). A total score ranging from 0 to 6 was obtained by summing up the answers on the three items, a score of 4 or more indicating the presence of distress.

#### Anxiety/depression

The 12-item General Health Questionnaire (GHQ-12) was used to assess psychological symptoms related to anxiety/depression in the previous four weeks (e.g., ‘Have you recently felt under strain?’) (Goldberg et al., 1997). The GHQ-12 had been validated in several languages including English, French and Spanish (internal consistency: 0.7 – 0.9; criterion-related validity: sensitivity ≥ 0.70, specificity ≥ 0.75, Area Under ROC Curve ≥ 0.83) (Goldberg et al., 1997; Salama-Younes et al., 2009). Based on the traditional scoring system, a total score ranging from 0 to 12 was calculated by summing up the answers on the 12 items, with a score of 2 or more indicating signs of anxiety/depression (Area Under Curve = 0.88) (Goldberg et al., 1997).

#### Sleeping disturbance

Based on the PROMIS (short form), sleep disturbance in the previous four weeks was assessed through two single questions (e.g., ‘Have you recently had some problem to sleep?’) scored on a 4-point scale (0 for favourable answers, 1 for unfavourable answers) (Buysse et al., 2010; Yu et al., 2011). The PROMIS had been validated in several languages (for detailed information, see www.nihpromis.org) including English, French and Spanish (internal consistency: >0.9; construct validity: product-moment correlations ≥ 0.96) (Buysse et al., 2010; Yu et al., 2011). A total score ranging from 0 to 2 was obtained by summing up the answers to the two questions, a score of 1 or more indicating the presence of sleep disturbance.

#### Adverse alcohol behaviour

The current level of alcohol consumption was evaluated using the 3-item AUDIT-C (e.g., ‘How many standard drinks containing alcohol do you have on a typical day?’) (Dawson et al., 2005). The AUDIT-C had been validated in several languages including English, French and Spanish (test-retest coefficients: 0.6 – 0.9; criterion-related validity: Area Under ROC Curve 0.70 – 0.97) (Dawson et al., 2005; De Meneses-Gaya et al., 2009). A total score ranging from 0 to 12 was obtained by summing up the answers on the three items, a score of 5 or more indicating the presence of adverse alcohol behaviour.

#### Smoking

Current smoking behaviour was assessed with a single question (e.g., ‘Do you smoke?’) (yes or no).

#### Adverse nutrition behaviour

Current eating habits were examined using four statements validated in English and Dutch (e.g., ‘I eat regularly throughout the day’), each to be answered by how many days per week (from 0 to 7) this is the case (Van der Veer et al., 2011). Consuming healthy meals less than five days per week and eating regularly throughout the day less than three days per week and having breakfast before 10:30 less than three days per week and having a final meal before 20:30 less than three days per week was reported as adverse nutrition behaviour.

### Stressors

#### Severe injuries

the number of severe injuries during a professional career was examined with a single question (e.g., ‘How many severe injuries have you had so far during your professional career?’). In our study, severe injury was defined as one that occurred during team activities and led to either training or match absence for more than 28 days (Fuller et al., 2006).

#### Surgeries

the number of surgeries undergone during professional career was examined with a single question (e.g., ‘How many surgeries have you had so far during your professional career?’).

#### Life events (LE)

Based on the validated Social Athletic Readjustment Rating Scale, the occurrence of life events (e.g., ‘Death of the spouse’, ‘Change in the financial state’) either in the previous six months (LE<6) or longer than six months ago (LE>6) was explored by 13 single questions (yes or no) (Bramwell et al., 1975). Two scores (LE<6 and LE>6) were calculated by summing up the life events occurred.

#### Career dissatisfaction

Professional soccer career dissatisfaction was explored through the validated Greenhaus scale (e.g., ‘I am satisfied with the success I have achieved in my career’) (5 items on a 5-point scale) (Greenhaus et al., 1990). A total score ranging from 5 to 25 was obtained by summing up the answers to the five items, a lower score indicating a higher level of dissatisfaction.

### Procedures

Based on the variables included in the study, an electronic questionnaire available in English, French, Japanese and Spanish was set-up. The following descriptive variables were also included: age, body height, body mass, duration of professional career, the level of play, a playing position, and the educational level. In order to guarantee the strict confidentiality of the responses, no personal identifiable information was included in the questionnaire. The World Players’ Union (FIFPro) – representing more than 65,000 professional players worldwide – asked the national players’ unions in Belgium, Chile, Finland, France, Japan, Norway, Paraguay, Peru, Spain, Sweden and Switzerland to select at random (simple random sample) potential participants from their members. Information about the purpose and procedures of the study was sent per email to potential participants by their national players’ unions. If interested in participating in the study, participants gave their informed consent and were asked to anonymously fill in the electronic questionnaire (FluidSurveys^TM^) within two weeks. Reminders were sent per email after two and four weeks. Once completed (around 15 minutes was needed), the electronic questionnaires were saved automatically on a secured electronic server. Players participated voluntarily in the study and did not receive any reward for their participation. Questionnaires were distributed online in April, May, August and September 2014.

### Data analyses

All data analyses were performed using the statistical software IBM SPSS Statistics 22.0 for Windows. Only questionnaires sufficiently completed were eligible for analyses: 50% of the descriptive variables and 50% of the outcome measures needed to be completed. Descriptive analyses (mean, standard deviation, frequency, range) were performed for the different descriptive variables, outcome measures and determinants involved. Prevalence of symptoms of CMD (distress, anxiety/depression, sleep disturbance) and adverse health behaviours (adverse alcohol, smoking and nutrition behaviours) was calculated, using the Wald method (sample size of more than 150) for 95% confidence interval (95% CI) (Portney and Watkins, 2008). Correlation coefficients (Spearman, Rank Biserial) were used to explore the direction and relative strength of the potential relationship between determinants (the number of severe injuries, the number of surgeries, the number of life events, the level of career dissatisfaction) and outcomes measures (Portney and Watkins, 2008). Univariate logistic regression analyses expressed as odds ratio (OR) and the related 95% confidence interval (95% CI) were performed to explain potential relationships between determinants occurred over the past years (the number of severe injuries, the number of surgeries, the number of life events, the level of career dissatisfaction) and the presence/absence of the outcomes measures under study at baseline (Portney and Watkins, 2008).

## Results

### Participants

Eleven national players’ unions contacted 1785 male professional soccer players, 661 of whom gave their written informed consent and filled in at least parts of the questionnaire (response rate: 37%). As 54 questionnaires were excluded from the analysis as they were insufficiently completed, 607 professional players were involved in the analyses. The participants were on average 27 years old, and 55% were playing in the highest leagues of their countries. During their career (mean duration of 7.8 years), the players had already incurred on average 2.2 severe injuries (SD=2.4), and had had 1.1 surgeries (SD=1.4). All characteristics of the participants are presented in Table 1.

#### Prevalence of symptoms of common mental disorders and adverse health behaviours

Prevalence of symptoms of CMD and adverse health behaviours among professional soccer players ranged from 4% for smoking and 9% for adverse alcohol behaviour to 38% for anxiety/depression and 58% for adverse nutrition behaviour. All prevalence rates are presented in Table 2.

#### Correlations and associations

Statistically significant correlations were found between stressors (a higher number of severe injuries, a higher number of life events and a higher level of career dissatisfaction) and symptoms of CMD and adverse health behaviours (except smoking). A higher number of severe injuries was associated with distress (OR=1.2; 95%CI 1.0–1.3) and sleeping disturbance (OR=1.1; 95%CI 1.0–1.2). A higher number of life events in the previous six months was associated with distress (OR=1.3; 95%CI 1.2–1.5), sleeping disturbance (OR=1.3; 95%CI 1.1–1.5) and adverse alcohol behaviour (OR=1.4; 95%CI 1.1–1.7). A higher level of career dissatisfaction was associated with distress (OR=0.9; 95%CI 0.9–1.0), anxiety/depression (OR=0.9; 95%CI 0.9–1.0) and adverse nutrition behaviour (OR=1.0; 95%CI 0.9–1.0). The direction and relative strength of relationships as well as associations between determinants (stressors) and outcomes measures are presented in Table 3.

## Discussion

The main finding of the present study was that prevalence of symptoms of CMD and adverse health behaviours among a sample of professional soccer players ranged from 4% for smoking and 9% for adverse alcohol behaviour to 38% for anxiety/depression and 58% for adverse nutrition behaviour. Also, significant associations were found for a higher number of severe injuries with distress, anxiety/depression, sleeping disturbance and adverse alcohol behaviour, for a higher number of life events with distress, sleeping disturbance, adverse alcohol behaviour and smoking, and for a higher level of career dissatisfaction with distress, anxiety/depression and adverse nutrition behaviour. Statistically significant correlations (p<0.01) were found for a higher number of severe injuries and a higher level of career dissatisfaction with most symptoms of CMD and adverse health behaviours (except smoking).

### Perspective of the findings

As far as the authors know, the present study is the first international study to explore symptoms of CMD and adverse health behaviours in professional soccer in such a large group of professional soccer players. In 2013, the World Players’ Union (FIFPro) conducted a preliminary study on the mental and psychosocial health problems among a smaller sample of 149 professional players from Australia, Ireland, the Netherlands, New Zealand, Scotland and the United States (Gouttebarge et al., 2015a). In their cross-sectional study, the authors found that prevalence of mental and psychosocial health problems was 10% for distress, 19% for adverse alcohol behaviour, and 26% for anxiety/depression as well as for adverse nutrition behaviour (Gouttebarge et al., 2015a). Most of the prevalence rates found in the present study are higher than in the FIFPro’s first study, reaching up to 38% for anxiety/depression and 58% for adverse nutrition behaviour. The higher prevalence of distress and anxiety/depression symptoms observed in the present study might be due to the recruitment period (April, May, August and September) involving the in-between seasons period, known to be difficult for many players because of the prevailing uncertainty of the continuation of their contract or transfer to a new club. In the present study, the occurrence of symptoms of distress, anxiety/depression, sleeping disturbance and adverse nutrition behaviour was associated with severe injuries in the previous years, life events taking place in the previous six months, and career dissatisfaction. This is in line with the results from the FIFPro’s preliminary study (Gouttebarge et al., 2015a). Consequently, one might suggest that monitoring the occurrence of these stressors during a professional career might be a good step towards identifying players predisposed for symptoms of CMD at an early stage in order to prevent these symptoms to develop into a serious mental disorder.

In professional, i.e. elite, athletes from other sport disciplines, studies about CMD are scarce. A recent study by Gulliver et al. (2015) explored the prevalence of symptoms of general psychological distress and CMD among Australian elite athletes. The authors found that around 45% of athletes were experiencing symptoms of at least one of the mental health problems (such as anxiety, depression or distress) assessed (Gulliver et al., 2015). In another recent study involving more than 2000 young and adult French Olympics athletes, 17% of them reported having encountered mental problems in the past (Schaal et al., 2011). In other studies using the same scales for most outcomes measures, prevalence of anxiety/depression was found to range from 13 to 19% in Australia (general population), from 17 to 21% in Denmark (practice population), and from 17 to 25% in the Netherlands (general and practice population, young male employees) (Korten and Henderson, 2000; Bültmann et al., 2002; Lynge et al., 2003; Verhaak et al., 2005). Prevalence of distress in both young and older working populations was reported to range from 5 to 18%, while burnout ranged from 5 to 7% in medical residents and physicians (Bültmann et al., 2002; Ruitenburg et al., 2012). Consequently, the findings of our study suggest that symptoms of CMD might be more prevalent among professional soccer players than in the general population or elite athletes from other sport disciplines. However, a comparison with these studies is limited since different instruments were used to assess outcome measures related to mental health problems to those used in our study.

### Methodological considerations

A limitation of the present study is the cross-sectional design that does not allow the establishment of a casual relationship between determinants, i.e. stressors and the outcome measures under study (Portney and Watkins, 2008). Another limitation might be related to the selection of the participants. Even if random sampling strategy was communicated to the unions in order to ensure that the participants were representative of the target population, this procedure was blind to the researcher for privacy and confidentiality reasons. Consequently, nonresponse analysis could not be conducted. Being higher than in the FIFPro’s first study (29%), the response rate in the present study was only 37%, which limits the generalizability of the findings. Moreover, since all countries were not equally represented in the study group, prevalence per country could not be calculated. Another potential limitation worth mentioning is that other determinants for symptoms of CMD such as family history of mental disorders were not included in our study. A last potential limitation is related to the instruments used in the present study: the translation of several scales into Japanese has not yet been validated, the questionnaire was not administered in the native language of some participants, the use of a single question to explore smoking, the lack of insight in the nutritional composition of meals consumed, and the use of self-report for the assessment of symptoms of CMD. We are well aware that a clinical instrument offers a more valid diagnostic tool to a given pathology. However, the choice of self-reported instruments to assess symptoms of CMD remains the most feasible one, especially with regard to the international character of the study.

The strength of the present study is that it investigated a new sensitive health aspect in a large group of elite players. In contrast to physical health, mental health still remains a taboo in professional soccer. This second study of FIFPro should contribute to raising of self-awareness of all stakeholders in professional soccer about the potential problems related to CMD among players. A second additional strength of our study is the number of participants included. Despite the fact that the 600 participants involved in the present study represent 1% of the total population of professional soccer players worldwide, the large sample of respondents allows us to gain a good primary insight into the extent of mental health problems in professional soccer. Furthermore, such an epidemiological study is a necessary first step to proposing adequate preventive and supportive measures aiming to protect player’s health and safety (Van Mechelen et al., 1992).

#### Implications

The findings of the current study might justify a multidisciplinary approach to a severely injured soccer player. After surgery for a severe injury, the responsible team physician as well as the orthopaedic surgeon performing the surgery and coordinating the rehabilitation program, should be aware of the potential occurrence of symptoms of CMD and adverse health behaviours accompanying such a situation. This awareness might allow (i) the early treatment of such disorders, (ii) a better and safer return to sports, and (iii) the application of supportive measures to prevent the development of severe mental disorders on the long term.

In terms of preventive and supportive measures, a recent study showed that young elite athletes reported that stigma was the most important perceived barrier to seeking help for CMD (Gulliver et al., 2012a). Another barrier was the lack of mental health literacy about such a topic, confirming the need mentioned previously by both current and former players as well as club physicians for proper attention to be paid to the psychosocial aspects of health (Gulliver et al., 2012a; Akturk et al., 2014). Consequently, raising the self-awareness about CMD that might occur during the career of professional players is a logical next step and should be the minimum standard. The future challenge for stakeholders in professional soccer, especially player’s unions, is also to develop and implement evidence-based interventions aiming to improve the mental health of players (Gouttebarge and Aoki, 2014). Therefore, interventions based on a self-management approach might be most appropriate in order to engage activities that protect and promote mental health and to monitor and manage symptoms of CMD and their impacts on functioning, emotions and interpersonal relationships (Barlow et al., 2002). Especially online interventions that can facilitate mental health help-seeking showed promise for decreasing stigma for CMD among elite athletes and for increasing their knowledge of these health conditions (Gulliver et al., 2012b). Such an approach should be explored in professional soccer.

## Conclusion

High prevalence of symptoms of CMD and adverse health behaviours was found among professional soccer players. Raising the self-awareness of professional players about CMD, facilitating the access to adequate treatment as well as developing and implementing evidence-based preventive measures should be prioritized by the different stakeholders within professional soccer. Future research in professional soccer should establish prevalence data in different representative samples from different cultural backgrounds, analyse risk factors using a prospective design, and evaluate the effects of interventions to prevent and/or treat mental and behavioural illness in football players.

## Figures and Tables

**Table 1:** Characteristics of the professional soccer players (N = 607).

Variables		
Age in years; mean (SD)	26.8	± 4.4
Body height in cm; mean (SD)	181.1	± 7.3
Body mass in kg; mean (SD)	76.8	± 8.1
Duration football career in years; mean (SD)	7.8	± 4.4
Top league players; N (%)	330	(54.4)
Field position; N (%)		
Goalkeeper	82	(13.5)
Defender	231	(38.1)
Midfielder	194	(31.9)
Forward	100	(16.5)
Educational level; N (%)		
No schooling completed	9	(1.5)
Nursery/Elementary school	19	(3.1)
High school	318	(52.4)
Vocational/technical school	74	(12.2)
College, university or equivalent	187	(30.8)
Severe injuries; mean (SD)	2.2	(2.4)
Surgeries; mean (SD)	1.1	(1.4)
LE<6; mean (min - max)	1.2	(0 – 9)
LE>6; mean (min - max)	2.0	(0 – 14)
Career dissatisfaction; mean (SD)	11.5	(3.7)

N, number of participants; SD, standard deviation; cm, centimetres; kg, kilograms; min, minimum; max, maximum; LE<6, life events in the previous 6 months; LE>6, life events longer than 6 months

**Table 2:** Prevalence of symptoms of common mental disorders among professional soccer players.

	N	Prevalence (95% CI)	
Distress^1^	81/548	14.8	(11.8–17.8)
Anxiety/depression^1^	187/493	37.9	(33.7–42.2)
Sleeping disturbance^1^	128/548	23.4	(19.8–26.9)
Adverse alcohol behaviour^2^	50/530	9.4	(7.0–11.9)
Smoking^2^	20/530	3.8	(2.2–5.4)
Adverse nutrition behaviours^2^	308/530	58.1	(53.9–62.3)

N, number of participants; CI, confidence interval;

1 1-month prevalence;

2 point prevalence

**Table 3:** Correlations (Pearson or Point Biserial) and associations (odds ratio and 95% confidence interval) between determinants and symptoms of common mental disorders among professional soccer players.

	Distress	Anxiety/depression	Sleeping disturbance	Adverse alcohol behaviour	Smoking	Adverse nutrition behaviour
Severe injuries	0.15**	0.13**	0.07	0.13*	−0.05	−0.03
	1.2 (1.0–1.3)**	1.0 (1.0–1.1)	1.1 (1.0–1.2)**	1.0 (0.9–1.2)	0.9 (0.7–1.2)	1.1 (1.0–1.2)
Surgeries	0.07	0.06	−0.02	0.15**	−0.02	−0.14**
	0.9 (0.7–1.1)	1.0 (0.8–1.1)	0.9 (0.8–1.1)	1.0 (0.8–1.3)	0.8 (0.5–1.3)	0.8 (0.7–0.9)**
LE<6	0.09*	0.08	0.09*	0.09*	0.05	0.05
	1.3 (1.2–1.5)**	1.1 (1.0–1.3)	1.3 (1.1–1.5)**	1.2 (1.0–1.5)*	1.4 (1.1–1.7)*	0.9 (0.8–1.1)
LE>6	0.09*	0.01	0.07	0.07	0.05	0.02
	1.1 (1.0–1.2)	1.0 (0.9–1.1)	1.0 (0.9–1.1)	1.2 (1.0–1.3)*	1.3 (1.0–1.5)*	1.0 (1.0–1.1)
Career dissatisfaction	−0.16**	−0.18**	−0.06	−0.09*	0.01	−0.12*
	0.9 (0.9–1.0)**	0.9 (0.9–1.0)**	1.0 (1.0–1.1)	1.0 (0.9–1.1)	1.0 (0.9–1.1)	1.0 (0.9–1.0)*

N, number of participants;

* p<0.05;

** p < 0.01;

LE<6, life events in the previous 6 months; LE>6, life events longer than 6 months
